# Short-term intervention complemented by wearable technology improves Trichotillomania – A naturalistic single-case report

**DOI:** 10.3389/fpsyg.2023.1071532

**Published:** 2023-09-04

**Authors:** Konstantin W. Leibinger, Eileen Murray, Steffen Aschenbrenner, Jennifer Randerath

**Affiliations:** ^1^Department of Psychology, University of Konstanz, Konstanz, Germany; ^2^Psychotherapy Training Center Bodensee (apb), Konstanz, Germany; ^3^Section for Clinical Psychology and Neuropsychology, Department for Psychiatry and Psychotherapy, SRH Klinikum Karlsbad-Langensteinbach, Karlsbad, Germany; ^4^Lurija Institute for Rehabilitation Science and Health Research, Kliniken Schmieder, Allensbach, Germany; ^5^Outpatient Unit for Research, Teaching and Practice, Faculty of Psychology, University of Vienna, Vienna, Austria

**Keywords:** Trichotillomania, single-case report, habit-reversal training, wearable technology, wearable device, naturalistic psychotherapy research

## Abstract

There is a growing interest in using wearable technology for the treatment of *body-focused repetitive behaviors* (BFRBs), such as Trichotillomania. Yet, to our knowledge, few studies address the applicability and use of wearable technology as a therapeutic element in more naturalistic situations. Here we would like to introduce its potential use combined with a Habit-Reversal Training in a single-case experimental design. In practice, individuals with BFRBs frequently show complex constellations of psychiatric disorders. Accordingly, the here presented participant was diagnosed with Trichotillomania as well as comorbid ADHD and examination phobia. The participant was offered to wear an unobtrusive and user-friendly vibration device that sent an alarm when her critical hairpulling behaviors occurred. The complementing Habit-Reversal Training included an *Awareness Training* supported by the vibration alarm of the wearable device. It further included a *Competing Response Training* by learning benign behaviors that could replace the hairpulling behavior. The frequency of hairpulling episodes was assessed using daily self-reports and by using the monitoring function of the wearable device. The intervention procedure was implemented into the participant’s everyday life and evaluated over the course of 214 days. The results indicated a significant reduction in the daily episodes of hair pulling. Our preliminary findings suggest that the here applied intervention has the potential to effectively treat Trichotillomania in individuals with comorbid disorders in psychotherapeutic outpatient care. Certainly, group-studies will need to further validate the approach’s effectiveness.

## Introduction

Trichotillomania is characterized by repeated hair pulling and unsuccessful attempts to decrease or stop the behavior that leads to (significant) hair loss. It is commonly associated with negative affective states like boredom, tension, or depressive mood (Duke et al., 2009) and can affect any hairy parts of the body. The symptoms can lead to substantial distress and health problems such as depression or anxiety (Stemberger et al., 2000; Deckersbach et al., 2003) or trichobezoar (Snorrason et al., 2021). The prevalence of Trichotillomania ranges from 0.6 to 15.3% (Mansueto and Rogers, 2012). Grant and Chamberlain (2022) reported that less than one-quarter of people with lifetime trichotillomania appear to experience natural recovery after 10.0 years. Of its multiple comorbidities, the attention-deficit and hyperactivity disorder frequently cooccurs (ADHD, in about 15–30%), and its co-occurrence goes along with significantly lower rates of natural recovery. A recent study by Chesivoir et al. (2022) with a large group of persons with Trichotillomania and ADHD showed that heightened impulsivity appeared to be a shared symptom, but the severity of ADHD or Trichotillomania was not correlated, and Trichotillomania severity was unaffected by stimulant ADHD medications.

Thus far Habit-Reversal Training (HRT) has the strongest evidence for treating Trichotillomania effectively (Farhat et al., 2020). The HRT intervention consists of several treatment components, out of which Awareness Training and Competing Response Training are the most important (Woods and Miltenberger, 1995). Many individuals are not aware of the occurrence of their BFRB (du Toit et al., 2001). During the Awareness Training participants take note of the movements of the BFRB (Azrin and Nunn, 1973). In the Competing Response Training, the therapist supports the participant to identify a behavior to replace the BFRB, which should be benign, and accepted by the participants and their social environment (Woods and Twohig, 2001). The competing response should be implemented immediately after each occurrence of the BFRB for a total of one minute (Miltenberger and Fuqua, 1985). However, maintaining awareness of a BFRB can be difficult while dealing with daily life tasks, such as studying.

Wearable technologies can increase the awareness of BFRBs by warning the individual each time the BFRB occurs (e.g., via vibration alert). According to Stricker et al. (2001) two different mechanisms are conceivable: (1) wearable technology leads to increased awareness of the BFRB and thereby reduces it and (2) the frequency of the BFRB is reduced due to alerts of the wearable technology via negative reinforcement. Up to now, the amount of studies examining the application of wearable technologies is still sparse. So far, studies display a lack of generalizability since the treatment measures analyzed were always obtained from laboratory settings. In addition, most studies thus far have been limited to the application of custom-made wearable technology, which can be difficult to access. A recently published pilot study by Stiede et al. (2022) implementing the purchasable “keen” device (HabitAware Inc., 2020) and app for 4 weeks in 10 persons with Trichotillomania reported high usability, acceptability, and perceived efficacy of the system with respect to increasing awareness and reducing hair pulling. This appears encouraging. The authors mentioned the limitations of the study being its short length and lack of follow-up.

We chose the research methodology of a single-case experimental design for its known advantages as an exploratory method: to account for complexity, grounded in and applicable to real-life, contemporary human situations and to provide in-depth relevant data (Krusenvik, 2016; Maric and van der Werff, 2020; Paparini et al., 2021). For our naturalistic case study, an individual with Trichotillomania and comorbid ADHD and examination phobia was recruited in an outpatient psychotherapy practice. The current study’s core aims were (1) to pilot test a purchasable wearable vibrating device and (2) to apply and assess the device over a longer period within a naturalistic context:

The purchased bracelet [“keen” by HabitAware Inc. (2020)] looked like a common fitness watch, appeared user-friendly and unobtrusive, and could be programmed to vibrate when particular movements were carried out. For the treatment of Trichotillomania, the device was combined with a simplified HRT. The procedure included a baseline-phase (implementation of the HRT, short-term psychotherapy and activation of the wearable device reacting to one relevant movement of Trichotillomania) and an experimental phase (full activation of the wearable device). The period for collecting the frequency of hair pulling events during natural, everyday situations lasted 214 days and originated from self-reports of the participant: As soon as the specified behavior occurred, the participant reacted with a recordable button press on the wearable device and subsequently carried out a competing response. Please note, after the experimental phase, the participant refused to stop using the wearable device. Therefore, implementing an additional baseline-phase as a follow-up assessment after the experimental phase was not possible.

In line with previous studies that implemented wearable technology into HRT (Stricker et al., 2001), we expect that the frequency of hair pulling during the baseline phase remains unchanged, because the wearable device is not fully activated during this phase. We hypothesized an immediate drop in the frequency of the hair pulling with the beginning of the experimental phase.

## Methods

Persons at the Psychotherapy Training Center Bodensee (apb) were informed about the opportunity to participate in the current study via brochures. Inclusion criteria consisted of fluency in German and reports of clinically relevant BFRBs according to ICD-10. The study corresponded to the Declaration of Helsinki and was approved by the Ethics Committee of the University of Constance (#15/2020). Please note: our manual (Randerath and Leibinger, 2023) with more details on how to conduct the intervention has been made available via the University of Konstanz’s institutional repository: https://doi.org/10.48787/kops/352-2-bnp1hvnzrrvr2.

### Participant

The participant gave written informed consent. There was no financial compensation for study participation. Two female individuals participated. However, in the Method and Result section, we will only focus on one participant. In the discussion we will address why the second individual opted out before the intervention started.

The 23-year-old participant was diagnosed in 2019 with Trichotillomania (F63.3) according to ICD-10 (Therapist and Supervisor opinions). The Trichotillomania symptoms had been present for at least 4 years. She had a comorbid ADHD disorder (F90.2, combined type; showing symptoms of inattention and hyperactivity) and examination phobia (F40.2: Other situational type phobia) according to ICD-10. In order to affirm the diagnosis of ADHD, we applied the HASE (Rösler, 2008).

The participant defined the symptoms of her Trichotillomania as “touching and pulling strands of hair with two or more fingers.” She reported that hair pulling increased during stressful periods (before writing exams) and occurred especially when studying. She reported shame about pulling hair and sadness about significant hair loss. The participant showed a picture with long, tight curly hair from before the illness. At the beginning of the study, she had short hair with bald spots visible on her left head area. Ruminating, anxiety and impulsivity had been reduced with the conventional treatment of 37 units cognitive behavioral therapy, but had not been completely eradicated. Since unit 27 the participant took 10 mg of Methylphenidate (Medikinet) as prescribed from her physician. She described her concentration to be improved. The participant stopped her conventional treatment for the time of a temporary employment abroad, going along with the length of the current study.

Day 1 to day 10 from study onset marked the timespan of baseline phase, which was combined with an additional psychotherapeutic short-term intervention. The experimental phase afterwards extended from day 11 to day 214. From day eleven to day 164, the participant spent several months abroad. During the entire experimental phase, she did not receive psychotherapeutic treatment. At the end of the study, on day 210, conventional psychotherapy was resumed. While participating in the study, the participant took 10 mg of Methylphenidate (Medikinet) per day to alleviate her ADHD. She decided to attach the wearable device to her non-dominant left arm.

### Materials and methods

#### Material and intervention

For our HRT a size M wearable “keen” device from HabitAware Inc. (2020) was combined with two treatment components: 1. *Awareness Training* and 2. *Competing Response Training*. For performing the competing responses, the participant received a carry-on box with different objects to choose from.

### Measures and analysis

We conducted a single-case AB phase design: The baseline-phase (A) covered a total of 10 days and included a simplified HRT. The wearable device responded only to one specific movement of the BFRB during the baseline. The baseline was accompanied by two more psychotherapeutic sessions conducted by the participant ‘s therapist, who was in advanced psychotherapy training. In these sessions the treatment opportunity and the upcoming stay abroad and plans for resuming psychotherapy afterwards were reflected. With the beginning of the experimental phase (B), the wearable device was fully activated by adding additional movements of the BFRB to the motion detection. In total five different movements of the BFRB were defined. This marked the beginning of the experimental phase, which included 214 days (August 2020 to March 2021), coinciding with the participant’s move to a different place and interruption of her conventional treatment.

*Hair pulling per day* was used as the dependent variable and comprised all the occasions the participant pressed the button of the wearable device over the course of a single day. The participant was asked to answer questions of a daily log at the end of each day. First, she had to indicate whether she wore the device on the respective day. Next, she indicated her mood and tension for three time points of the day on a five-point Likert scale. A mean score for each, mood and tension, was calculated per day. Finally, the participant was asked to shortly describe her daily activities in an open-question format. In order to measure potential change of responsiveness, TAP-Alertness was administered to the participant before and right after attending the intervention (Zimmermann and Fimm, 2017).

The participant evaluated the intervention by use of the German Therapy Experience Questionnaire. In the results we additionally refer to published TEQ data of a sample of 54 outpatients and inpatients attending and evaluating psychotherapeutic treatment (Linden et al., 2008). In order to receive detailed feedback about the intervention, results of the questionnaire were discussed with the participant afterwards.

The episodes of hair pulling on all 10 days during baseline phase, and 149 days of data from the experimental phase were included. On eight separate days over the course of the experimental phase, the participant did not submit any data on the frequency of hair pulling, and 26 separate days were excluded since the participant had not worn the wearable device on these days. A period of 21 consecutive days (day 42 to day 62) the participant did not use the wearable device in order to avoid being distracted by the vibration but focus on learning (see also discussion).

We displayed the frequency of hair pulling per day by using R version 4.1.0 (R Core Team, 2021) and the r packages “ggplot2” (Wickham, 2016), “readxl” (Wickham and Bryan, 2019) and “dplyr” (Wickham et al., 2021). Subsequently, graphs were interpreted by visual analysis (Kratochwill et al., 2010). The statistical analysis of the single case considered Tau-U indices to quantify trends within the baseline phase and trends within the experimental phase as well as changes between the baseline and the experimental phases (Brossart et al., 2018).

## Results

Tables with statistics and further assessments (TAP-Alertness, TEQ, HASE) are available in the Supplementary document.

### Visual analysis

**Figure 1:**
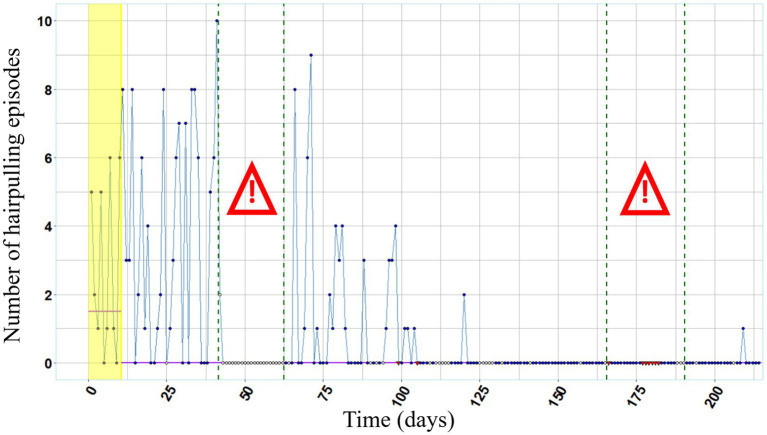
Frequency of hair pulling over the entire course of the study. Annotations. Red colored data points (missing data) and white colored data points (no use of the wearable device) were excluded from the analysis. Purple lines represent the median values of the frequency of hair pulling per day during baseline phase and experimental phase, respectively. The baseline phase is highlighted in yellow. Both exam periods are framed in green dashed lines and marked with the exclamation mark.

Figure 1 shows that hair pulling episodes occurred during both the baseline and the experimental phase. In the beginning of the experimental phase the maximum frequency of hair pulling episodes per day (N = 10 until day 31 of the experimental phase) exceeded the maximum frequency of hair pulling episodes per day in the baseline phase (N = 0 to N = 6 on 10 days in total). Over the course of the experimental phase daily hair pulling episodes slowly receded, and during the latter segment of the experimental phase hair pulling was nearly absent (0–1 episodes per day beginning from day 111 to day 204).

The examination periods were reconstructed using the entries of the daily log. During the first examination period of 21 days, we have no data available. During the second examination period of 25 days, we have data of in total 15 days available. Based on these data, hair pulling did not occur at any day.

The median of hair pulling frequency is 1.5 during the baseline phase and 0 during the experimental phase, indicating a reduction in hair pulling over the course of the experimental phase. The high variability in the frequency of hair pulling episodes decreases during the second half of the study.

### Trends and changes in baseline and experimental phase

While the Tau-U index does not indicate a significant trend in the baseline phase, it quantifies a significant negative trend in the experimental phase. The latter reflects a significant decrease in the frequency of hair pulling per day over the course of the experimental phase. Further, the Tau-U Index indicates a significant negative change between the baseline and experimental phase. Thus, the frequency of hair pulling was lower in the experimental phase compared to the baseline phase. According to the interpretation guidelines by Parker and Vannest (2009), this index (Tau-U = −0.4791, Z = −2.533, *p* = 0.011) is in the range of medium effect sizes (∣0.32–0.84∣).

### Self-reported mood and tension

**Figure 2:**
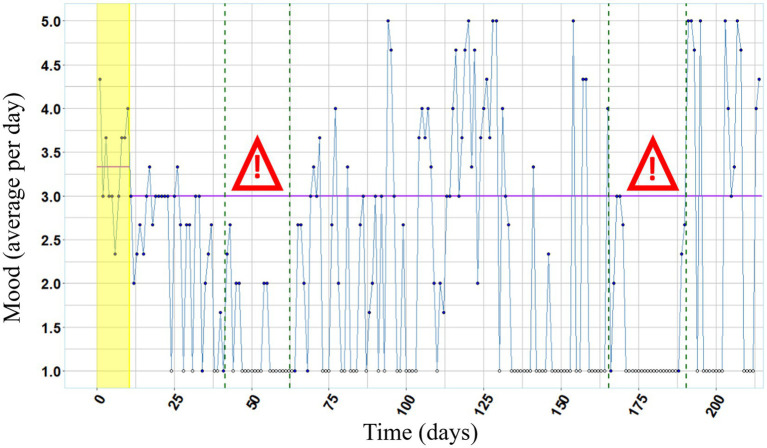
Mood over the course of the study. Annotations. White colored data points (missing data) were excluded from the analysis. Purple lines represent the median values of mood (1 = very bad, 2 = bad, 3 = neutral, 4 = good, 5 = very good) per day during baseline phase and experimental phase, respectively. Both examination periods are framed in green dashed lines and marked with the exclamation mark.

**Figure 3:**
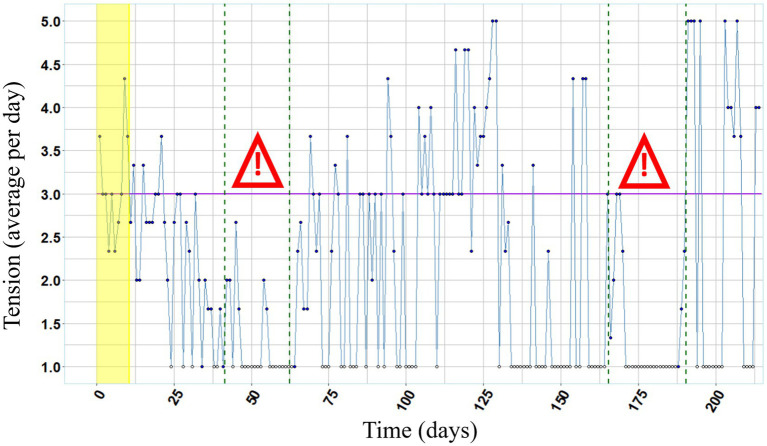
Tension over the course of the study. Annotations. White colored data points (missing data) were excluded from the analysis. Purple lines represent the median values of tension (1 = very tense, 2 = tense, 3 = neutral, 4 = relaxed, 5 = very relaxed) per day during baseline phase and experimental phase, respectively. Both examination periods are framed in green dashed lines and marked with the exclamation mark.

Figure 2 depicts self-reported mood and Figure 3 depicts self-reported tension over the entire course of the study. Lower values indicate bad mood and high tension, respectively. During both examination periods, mood as well as tension were below the respective median value of the experimental phase.

### TEQ

TEQ results are reported descriptively with comparative data of a psychiatric sample judging their respective therapeutic approach in brackets (Linden et al., 2008). The participant evaluated the approach positively (corresponds to lower scores with Min:1, Max: 5) in the following subscales: “Being informed about the therapy” (participant’s score = 1.17; Linden *et al*’s sample: M = 2.25; SD = 0.71); “Personal commitment to the therapy” (participant’s score = 1.13; Linden *et al*’s sample: M = 1.78; SD = 0.59); “Distrust and feelings of being at the mercy of the therapy” (participant’s score = 1.13; Linden *et al*’s sample: M = 1.40; SD = 0.35), “Fears and apprehensions of negative consequences of therapy” (participant’s score = 2.00; Linden *et al*’s sample: M = 1.41; SD = 0.46) and “Effects on one’s own competencies” (participant’s score = 1.86; Linden *et al*’s sample: M = 1.65; SD = 0.52).

The participant evaluated the approach toward the more negative end in the following subscale: “Effects of the therapy on social networks and everyday life” (participant’s score = 3.25; Linden *et al*’s sample: M = 2.59; SD = 0.77).

## Discussion

### Treatment effects

The magnitude of reduction in hair pulling between baseline– and experimental phase can be categorized as a medium effect size, and in the second half of the experimental phase hair pulling was nearly extinct. According to her self-reports, we can conclude that mood was lower and tension was higher than median during both examination periods, that possibly was amplified by her comorbid examination phobia. Before the intervention the participant indicated that hair pulling increased during stressful exam periods going along with empirical research revealing a higher occurrence of hair pulling in situations when participants reported depressive mood or tension (Duke et al., 2009). In line with the emotion regulation model (Roberts et al., 2013) it could be assumed that the execution of hair pulling is a mechanism to reduce such negative affective states. Therefore, the participant’s lack of hair pulling during the second stressful examination period, points to a meaningful treatment progress. Furthermore, the bald patches in her hair were hardly visible after the intervention. Consistent with existing research (e.g., Stricker et al., 2001; Himle et al., 2018), we demonstrated that the use of wearable technology in HRT can be effective also in a naturalistic setting by application of an unobtrusive and user-friendly wearable device to a person suffering from Trichotillomania with comorbidities of examination phobia and ADHD.

Phenomena such as reactivity or maturation might provide an alternative explanation for the reduction in hair pulling independent from the intervention (e.g., reactivity: a reduction in hair pulling due to daily assessment of the frequency of hair pulling episodes; maturation: natural reduction in hair pulling of the participant over time, independent from intervention) (Kratochwill et al., 2010; Grant and Chamberlain, 2022). However, the Tau-U index quantified no noticeable changes in hair pulling in the ten days before treatment onset, but a significant trend in the experimental phase and an overall change between baseline– and experimental phase. Further, at least a slight increase in hair pulling during the second examination period would be natural. But there was a lack of re-emergent hair pulling supporting the argument that the improvement can be related to the intervention. Additionally, speaking against a natural recovery, the participant’s reliance on wearing the vibrating wristband continued throughout the study. At the end of the study the participant reported that attempted pulling would reoccur in stressful situations when not wearing the wristband.

In contrast to previous studies in the field showing a typical drop of the behavior (Himle et al., 2008) our participant demonstrated a slow decrease of hair pulling behavior. Two different points may account for the rather gradual reduction. First, a sufficient intensity of aversive stimuli has been shown to be a prerequisite for an immediate and strong reduction of unfavorable behaviors (Williams et al., 1993). However, the current device only produces tolerable vibrations. Second, the participant demonstrates a reduced response inhibition that may have contributed to slowing down the progress. Consistent with the literature pointing to reduced response inhibition in individuals with ADHD (Jiang et al., 2018) deficits in response control in the TAP-Alertness test were evident in the participant before and after the intervention (See Supplementary document 2). The reduced response inhibition may contribute to hampering the suppression of hair pulling.

Being finally able to stop the unwanted impulsive behavior mostly independent from others (psychotherapeutic intervention only lasted eleven days and the participant decided for herself to use the device) may have created a source for self-efficacy, which also may have played a central role in the treatment success (Maddux and Kleiman, 2016). In the brief check-in a year after the intervention, the participant reported that she had decided to continue wearing the device. She further continued to use alternative behaviors (preferably the massage ball). She expressed that she could mostly rely on the ‘supportive control by the device’ which allowed her ‘to use and direct her attentional capacities toward other tasks that were more important to her than the unwanted behavior’. She reported no re-emergence while wearing the device. The device thus appeared to fulfill a compensatory function by strengthening awareness for her impulsive behavior.

### Acceptance and adherence

The participant reported that the device felt like a fitness watch and its rates of false alarms to be very low.

Hair pulling during the baseline phase was at medium frequency, followed by a higher frequency of hair pulling at the beginning of the experimental phase going along with the beginning of an exam-period. During the following 21 days missing data occurred. In a subsequent online-meeting, the participant reported that despite the known reoccurring loss of control with respect to hair pulling during exam-periods, she decided not to use the wearable device. The vibration of the wearable device distracted her while preparing for the exams (possibly amplified by her comorbid ADHD disorder). The time after the examination period was marked by a decrease in the frequency of hair pulling. Furthermore, for 24 separate days there was missing data during the experimental phase because the participant left the wearable device at home in order not to lose it (e.g., when she went for a journey). During these days, the participant did not report any hair pulling episodes in retrospect.

Results of the therapy evaluation questionnaire (TEQ) indicated that the participant felt sufficiently informed about the entire treatment period, felt personally committed and experienced the therapy without feelings of mistrust or abandonment. A still acceptable but not as positive evaluation was achieved in the subscale “fears of negative consequences”: The participant reported that she felt excluded and different compared to others, and she felt under high pressure not to pull her hair in situations when the vibrations of her wearable device were perceivable for others (e.g., when studying in a group). These feelings may be caused in particular by her tendency toward perfectionism and her fear of failure (e.g., examination phobia). For the participant, the subscale “positive impact of the therapy on social network and everyday life” was associated with both positive and negative aspects: On the one hand, the perceivable vibrations of her wearable device activated social resources (her partner reminded her to stop pulling hair when he heard the vibration alarm) on the other hand, her concentration decreased due to being distracted by the vibrations of the wearable device. This experience may have been amplified by her ADHD. The overall medium values on the subscale “positive consequences of the therapy on social network and everyday life” could be explained by the specific focus of the intervention on the behavioral level of the BFRB. Items of the questionnaire that refer to other levels important to psychotherapy (e.g.: “In therapy, I learn to deal better with feelings”) therefore were negated by the participant.

Thus, the participant demonstrated good acceptance and adherence with exceptions being linked to particular situations and to protective behavior securing the integrity of the wristband.

One female individual suffering from Dermatillomania (pathological skin picking) and comorbid ADHD had been recruited for our study but dropped out early. After the activation of the wearable device, she reported too many false alarms triggered by everyday movements (e.g., making a telephone call or drinking a cup of coffee), − although the researcher adapted the settings of the wearable device to movements of the person that were easier to discriminate from everyday movements and with movement sensitivity set to the lowest level. Because the device still gave too many false alarms the individual decided to discontinue. Thus, adequate performance of the movement sensor (sensitivity, specificity for a given behavior as well as technical functioning) appears essential for the effectiveness of the implemented wearable technology and may depend on the individual’s particular movement patterns. Presumably, the trajectories of hand movements of Dermatillomania are more difficult to discriminate from everyday hand movements by the movement sensor compared to the trajectories of hand movements related to Trichotillomania. This is in line with Stiede et al. (2022) who reported that the reliability of the device was an issue in their study as well, as some of their users reported experiencing false positives.

### Limitations and future prospects

The current promising results need to be interpreted by considering typical limitations of a single-case report (Lambert, 2005; Gustafsson, 2017) (e.g., placebo effects; the participant’s expectation for change, discussion about the BFRB, attention & warmth). In order to draw more valid inferences about the effectiveness of the wearable device, group studies including larger samples and randomized-controlled trials are required. Future studies should also consider including a longer baseline phase to better determine the influence of maturation, reactivity or placebo effects, and they should consider to inform participants that there can be particular situations during which adherence to wearing the wristband and performing the alternative actions may be difficult. Furthermore, inter-individual differences in experienced specificity of the wristband’s alarms need to be studied. Further, accompanying psychotherapeutic treatment may be recommendable in order to increase the perceived positive consequences of the therapy on social network and everyday life.

Summarized, our Trichotillomania case demonstrated overall good acceptance and adherence and clinically relevant improvement of her Trichotillomania symptoms. Single-case reports are an initial, cost-effective step to investigate the effects of new technologies. The present naturalistic single-case report has shown that the incorporation of wearable devices into HRT are a promising treatment approach for Trichotillomania.

## Data availability statement

The original contributions presented in the study are included in the article/Supplementary material, further inquiries can be directed to the corresponding author.

## Ethics statement

The studies involving humans were approved by University of Konstanz Ethics Committee. The studies were conducted in accordance with the local legislation and institutional requirements. The participants provided their written informed consent to participate in this study. Written informed consent was obtained from the individual(s) for the publication of any potentially identifiable images or data included in this article.

## Author contributions

KL and JR designed the study. KL implemented the device and training. KL analyzed the data. KL, JR, EM, and SA discussed the analyses and results and wrote the manuscript. All authors contributed to the article and approved the submitted version.

## Funding

This study was funded by an intersectional programme of the Zukunftskolleg at the University of Konstanz supported by the Excellence Strategy of the German Federal and State Governments at the University of Konstanz. The Open Access fee was covered by the University of Vienna.

## Conflict of interest

The authors declare that the research was conducted in the absence of any commercial or financial relationships that could be construed as a potential conflict of interest.

## Publisher’s note

All claims expressed in this article are solely those of the authors and do not necessarily represent those of their affiliated organizations, or those of the publisher, the editors and the reviewers. Any product that may be evaluated in this article, or claim that may be made by its manufacturer, is not guaranteed or endorsed by the publisher.

## Abbreviations

BFRB: body-focused repetitive behavior
HRT: habit-reversal training
