# Supplementary material for: Short-term intervention complemented by wearable technology improves Trichotillomania – A naturalistic single-case report
Source: Front Psychol. 2023 Sep 4;14:1071532. doi: 10.3389/fpsyg.2023.1071532 (PMC10507401; doi:10.3389/fpsyg.2023.1071532)
Supplement: Supplementary file 1 [file Data_Sheet_1.docx]

**Supplementary Material: Short-term intervention complemented by wearable technology improves Trichotillomania – a naturalistic single-case report**

Konstantin W. Leibinger, Eileen Murray, Steffen Aschenbrenner & Jennifer Randerath

**Content**

[**1.** **Study procedure** 2](#_Toc142905514)

[**1** **ADHD Assessment: HASE** 2](#_Toc142905515)

[**2** **Table 1: Results of the TAP-Alertness.** 2](#_Toc142905516)

[**3** **Table 2: Descriptive data of hair pulling episodes** 3](#_Toc142905517)

[**4** **Table 3: Calculated values of the Tau-U Indices**. 3](#_Toc142905518)

[**5** **Therapy Experience Questionnaire (TEF).** 3](#_Toc142905519)

[**6** **References** 4](#_Toc142905520)

# **Study procedure**


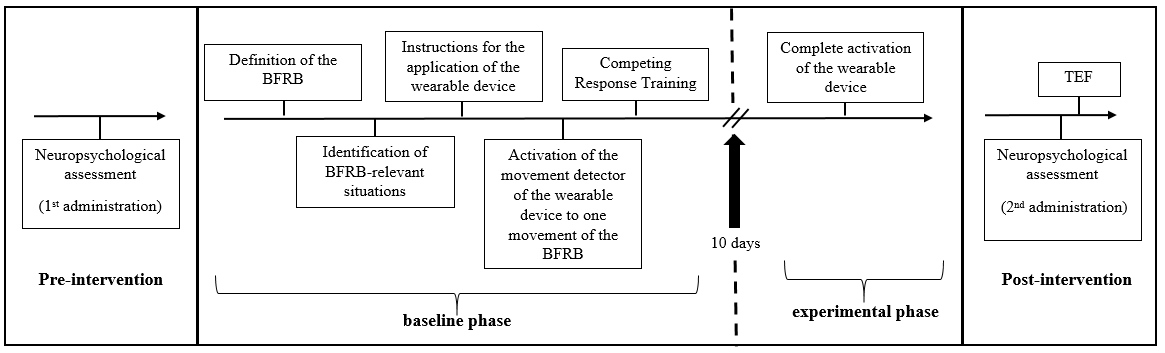
Study procedure. On day one, the simplified HRT was administered which encompassed (1) the definition of the *body-focused repetitive behaviors* (BFRB), (2) the identification of BFRB-relevant situations, (3) instructions for the application of the wearable device, (4) activation of the movement detector of the wearable device to one movement of the BFRB, and (5) the Competing Response Training. The entire baseline phase was accompanied by additional psychotherapeutic treatment. On day 11 after study onset, psychotherapy ended and the wearable device was completely activated to detect five different movements of the BFRB in total. TAP-Alertness was administered before and after the intervention. Treatment evaluation with the TEF was administered after the intervention.

# **ADHD Assessment: HASE**

The sum score of the WURS-K was 39, exceeding the cutoff-value of 30. In the ratings of the therapist using the ADHD-DC, five of nine criteria were met in the subscale “Inattention”, scoring below the cutoff-score of six criteria; four of five criteria were met in the subscale “Hyperactivity”, exceeding the cutoff-score of three criteria; two of four criteria were met in the subscale “Impulsivity”, exceeding the cutoff-score of zero criteria. Finally, the sum score of the ADHD-SB was above the critical cutoff-score (sum score = 32; cutoff-score = 18).

# **Table 1: Results of the TAP-Alertness.**

| **Condition:** | **Median reaction time (ms):** | **Percentile Rank:** | **Omissions:** | **Anticipations:** |
| --- | --- | --- | --- | --- |
| first administration (before intervention): | | | | |
| intrinsic alertness: | 261 | 24 | 1 | 0 |
| phasic alertness: | 578 | < 1 | 0 | 3 |
| second administration (after intervention): | | | | |
| intrinsic alertness: | 207 | 82 | 0 | 0 |
| phasic alertness: | 509 | < 1 | 0 | 0 |

The TAP-Alertness is a computerized neuropsychological test comprising the following two conditions: (1) the participant has to respond as fast as possible to a cross appearing at randomly varying time intervals on the monitor (tonic or intrinsic alertness); (2) the same cross is preceded by an auditive stimuli, again the participant has to respond to the cross only (phasic alertness). Prolonged responses (ms) or higher frequencies of anticipatory errors in the second condition compared to the first condition point to a problem in executive control (the ability to supress response to auditive stimuli). Differences in response times between pre and post intervention were classified with respect to the reported difference values that indicate significant change in the TAP-manual (Zimmermann & Fimm, 2017, p. A15).

Response times of the participant were inside the average range for the intrinsic alertness conditions, whereas response times in the phasic alertness condition were far below the average range. This pattern was consistent across both test administrations (pre-intervention and post-intervention). However, the differences in reaction times between pre-intervention and post-intervention exceeded the difference required for significance for intrinsic alertness (observerd difference: 54; difference required for significance: 21.808, p < 0.05) as well as for phasic alertness (observerd difference: 69; difference required for significance: 24.395, p < 0.05) pointing towards significantly shorter reaction times at post-intervention. Further, in the second administration she did not produce any anticipation errors, while in the first administration the participant reacted on the tone instead of waiting for the visual cue in three out of 43 trials.

# **Table 2: Descriptive data of hair pulling episodes**

The average frequency per day for the baseline and experimental phase.

| baseline phase | mean  median | 2.700  1.500 |
| --- | --- | --- |
|  | standard deviation | 2.497 |
|  | minimum | 0 |
|  | maximum | 6 |
| experimental phase | mean  median | 1.161  0.000 |
|  | standard deviation | 2.349 |
|  | minimum | 0 |
|  | maximum | 10 |

# **Table 3: Calculated values of the Tau-U Indices**.

| Index | Tau-U | SD Tau | Z | | p |
| --- | --- | --- | --- | --- | --- |
| trend in baseline phase | -0.067 | 0.249 | -0.268 | 0.788 | |
| trend in experimental phase | -0.333 | 0.055 | -6.027 | **0.000**** | |
| change in baseline- vs. experimentalphase | -0.4791 | 0.189 | -2.533 | **0.011*** | |

*p < 0.05; **p < 0.01

Autocorrelation can occur in single-case research due to the repeatedly performed measurements on the same subject (Singmann & Kellen, 2019). We chose the Tau-U indices (Parker, Vannest, Davis, & Sauber, 2011; http://www.singlecaseresearch.org/) since they are robust to autocorrelation.

# **Therapy Experience Questionnaire (TEF).**

The 40 items of the TEQ (TEQ; Linden, Lind, & Quosh, 2008) gather information about how the person experienced the psychological intervention on a 5-point Likert scale (do not agree at all (1); agree completely (5)). According to factor analysis, each item of the TEQ can be attributed to one of the following six subscales: (1) “) Fears and apprehensions of negative consequences of therapy”, (2) “Being informed about the therapy”, (3) “Effects of the therapy on social networks and everyday life”, (4) “Effects on one's own competencies”, (5) “Personal commitment to the therapy”, (6) “Distrust and feelings of being at the mercy of the therapy”. The calculated means of the subscales can range from one to five. Items asking about positive aspects of the intervention were inversed, so that low item values always represented a positive attitude towards the therapy. For each subscale, item means were calculated and compared to means of a psychiatric sample.

# **References**

Linden, M., Lind, A., & Quosh, C. (2008). Der Therapieerleben-Fragebogen (TeF) für Patienten und Angehörige. *Verhaltenstherapie, 18*(1), 35-42. doi:https://doi.org/10.1159/000117181

Parker, R. I., Vannest, K. J., Davis, J. L., & Sauber, S. B. (2011). Combining nonoverlap and trend for single-case research: Tau-U. *Behavior Therapy, 42*(2), 284-299.

Singmann, H., & Kellen, D. (2019). An introduction to mixed models for experimental psychology. In *New methods in cognitive psychology* (pp. 4-31): Routledge.

Vannest, K. J. P., R. I.; Gonen, O.; Adiguzel, T.;. (2016). Single Case Research: Web based calculators for SCR analysis. Retrieved from http://www.singlecaseresearch.org/

Zimmermann, P., & Fimm, B. (2017). TAP Testbatterie zur Aufmerksamkeitsprüfung Version 2.3.1: Vera Fimm, Psychologische Testsysteme.
